# Decompression sickness-induced skeletal muscle injury: an animal model and pathological analysis

**DOI:** 10.3389/fvets.2024.1431110

**Published:** 2024-09-12

**Authors:** Guanghua Chen, Yongbin Huang, Chunman Huang, Liwei Li, Jingqun Pang, Hongqiang Li, Wenxi Zhang

**Affiliations:** Orthopedic Center, Affiliated Hospital of Guangdong Medical University, Zhanjiang City, China

**Keywords:** decompression sickness, skeletal muscle injury, decompression-induced osteonecrosis, pathological analysis, animal model

## Abstract

**Aims:**

The primary objective of this investigation is to establish an animal model that accurately represents skeletal muscle injury as a consequence of decompression sickness. Additionally, this study aims to delineate the potential mechanisms underlying the development and progression of skeletal muscle damage associated with decompression sickness.

**Materials and methods:**

(1) In this research, rats were utilized as experimental models and subjected to 600 kPa pressure in an air medium for a duration of 60 min, followed by decompression at a consistent rate of 1.5 min to reach atmospheric pressure in order to establish an animal model for decompression injury. Assessment of decompression injury involved the observation of general symptoms and signs, as well as histopathological examination of lung tissue to determine the extent of damage in the pulmonary system of rats. (2) Building on the rat decompression injury model, we conducted pathological and serological examinations to assess the status of rat skeletal muscle. Additionally, we investigated the signaling mechanism of the TLR9-MyD88 pathway in mediating alterations in rat skeletal muscle resulting from decompression injury, and evaluated the effects of decompression injury on apoptosis in rat skeletal muscle.

**Results:**

Repeated decompression induces significant damage to skeletal muscle tissue, characterized by edema, fiber rupture, and atrophy. This process also leads to a transient elevation in creatine kinase (CK-MM) levels in rat serum, as well as an upregulation of proteins such as TLR9, MyD88, p38, and ERK in rat skeletal muscle tissue. Furthermore, repeated decompression results in a temporary increase in the transcription levels of Atrogen-1mRNA and MuRF-1mRNA in rat skeletal muscle tissue.

**Discussion:**

The decompression protocol applied in this study successfully induced decompression sickness in a rat model, leading to skeletal muscle damage that was consistent with the expected pathology of decompression injury. Despite the initial injury, the rats showed evidence of adaptation following prolonged exposure to decompression conditions.

## Introduction

The earliest documented cases of decompression sickness date back to 1841, when a substantial number of coal miners emerging from pressurized tunnel environments exhibited symptoms such as muscle cramps and pain. The manifestation of “the bends” typically occurred within 1 h following insufficient decompression. The prevalence of limb or joint pain in divers during ascent has generated considerable interest within the academic community. Researchers, including Spyros Peppas et al. (1) have suggested that nerve injury resulting from decompression bubbles—whether through direct stimulation or inflammation mediation—plays a pivotal role in the onset of physical discomfort and sensory disturbances. Nevertheless, the precise mechanisms remain incompletely understood. Decompression bubbles not only cause mechanical damage to tissues but also injure the vascular endothelium, thereby impairing blood circulation. These bubbles interact with blood components such as platelets and leukocytes, initiating inflammatory and procoagulant responses (1). Consequently, decompression sickness has the potential to cause tissue ischemia and spasmodic pain by compromising blood supply. Bigley et al. (2) detected increased expression of intercellular adhesion molecule-1 (ICAM-1) in the quadriceps femoris of Sprague–Dawley rats 24 h after decompression. Additionally, colleagues observed elevated levels of ICAM-1, E-selectin, and L-selectin in these tissues. Given that selectins are closely associated with tissue inflammation, it is conceivable that skeletal muscle damage resulting from decompression may trigger inflammatory responses. Studies (3) have documented instances of muscle damage in cetaceans exposed to conditions resembling decompression, suggesting that skeletal muscle injury during decompression may play a significant role in pain perception, independent of nerve or vascular damage.

Inspired by these findings, we propose the hypothesis that decompression sickness in rats may lead to localized skeletal muscle damage as a result of nitrogen bubble stimulation and vascular injury. Consequently, the objective of this research is to establish a rat model of decompression sickness and examine the histological alterations in skeletal muscle tissue during acute episodes of decompression sickness.

## Materials and methods

### Ethical approval

All protocols for this study have been approved by the Animal Experiment Ethics Committee of the Affiliated Hospital of Guangdong Medical University (approval number: AHGDMU-LAC-B-202308-0064).

### Materials

Anti-ERK (Abcam, Cambridge, MA, Cat.No.ab184699), anti-MyD88 (Abcam, Cambridge, MA, Cat.No.ab219413), anti-p38 (Abcam, Cambridge, MA, Cat.No.ab170099), and anti-TLR9 (SAB, Maryland, PA, Cat.No.53889).

### Experimental animals

Forty-eight male Sprague Dawley (SD) rats were obtained from Liaoning Changsheng Biotechnology Co., Ltd. All rats were 8 weeks old and weighed 300 ± 10 g at the time of the experiment. The animals were housed under specific pathogen-free (SPF) conditions, fed with standardized rat chow, and provided unrestricted access to drinking water. The environmental conditions maintained a temperature range of 20–24°C and relative humidity between 50 and 60%, with a 12-h light/dark cycle. All materials (including lids, food containers, water bottles, bedding, and water) were autoclaved prior to use. The rats were randomly divided into six groups using block randomization, facilitated by a computer-based random number generator (4). These groups included a blank control group (Control group, *n* = 8), a normal pressure group (Normal pressure group, *n* = 8), and a decompression group (Decompression group, *n* = 32). As per the experimental design, there was a 24-h interval between each decompression session, which occurred once or twice daily. The decompression group was subdivided into four subgroups: the one-day group (D1 group, *n* = 8), the two-day group (D2 group, *n* = 8), the three-day group (D3 group, *n* = 8), and the four-day group (D4 group, *n* = 8).

**Figure 1:**
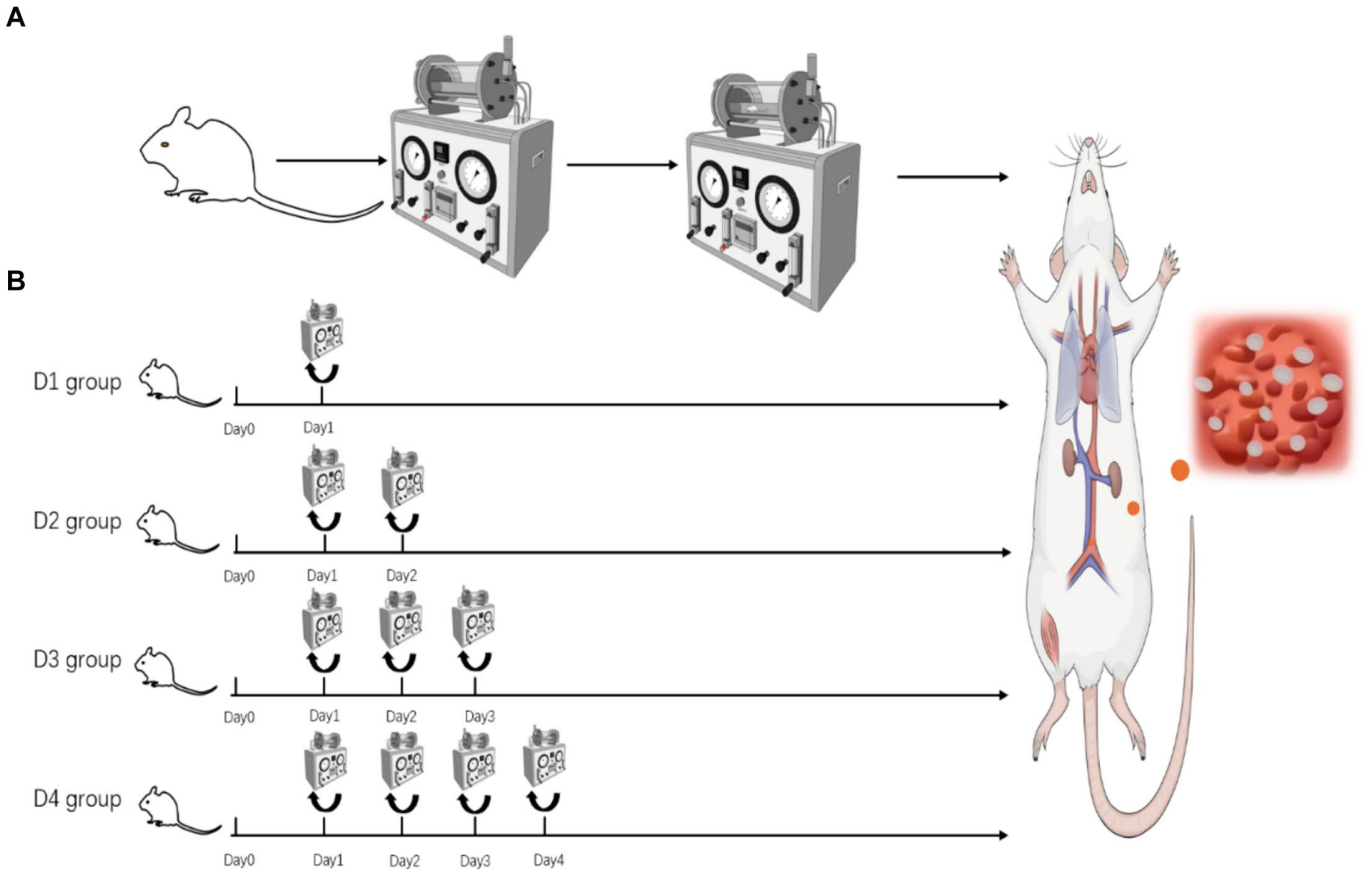
**(A)** Pressurization–decompression procedure flowchart. **(B)** Schematic diagram of rat grouping in the decompression group.

Rats in the control group were not exposed to the experimental chamber. Rats in the normal pressure group were introduced into and removed from the chamber under ambient atmospheric conditions, ensuring that factors such as time, temperature, and humidity within the chamber were comparable to those of the decompression group. Figure 1 illustrates the placement of rats in the decompression groups (D1, D2, D3, and D4) in a DWC150 animal experimental chamber and subjected to the following protocol: (1) The chamber was pressurized to 400 kPa at a rate of 100 kPa/min, followed by pressurization to 600 kPa at a rate of 150 kPa/min; (2) They were exposed to 600 kPa for 60 min with continuous ventilation (1 L/min), while monitoring oxygen (O2), carbon dioxide (CO2), temperature (maintained at 23–25°C), and relative humidity (65–75%); (3) Decompression occurred at a constant rate of 400 kPa/min until reaching normal atmospheric pressure. The D1 group underwent a single decompression session, the D2 group underwent two consecutive decompression sessions, the D3 group underwent three consecutive decompression sessions, and the D4 group underwent four consecutive decompression sessions. The rats’ behavior was observed immediately upon exiting the chamber. Three hours after the experimental intervention, rats in each group were euthanized by cervical dislocation, and anatomical samples were collected to detect changes in the structure and function of the lungs and skeletal muscles.

### Observation of gross behavior upon exiting the chamber

After decompression, house the rats in animal cages and observe their behavioral responses, including respiratory patterns, heart rate, and activity levels. The manifestation of symptoms such as scratching, impaired mobility, rapid or labored breathing, limb paralysis, or mortality signifies the onset of decompression sickness (DCS). Detailed records documenting the onset times and symptoms of the rats should be meticulously maintained, and both the incidence and mortality rates should be calculated. Animals that developed the aforementioned symptoms associated with decompression sickness were included in subsequent studies, which involved tests on serum, lung, skeletal muscle, and other tissues. Animals that died prematurely and for which behavioral and histological data could not be collected were excluded from further analyses.

### CK-MM assay

Three hours after leaving the chamber, the rats were euthanized by cervical dislocation, and blood was collected from the jugular vein. After incubation at 4°C for 12 h, the blood was centrifuged at 3,000 rpm for 10 min, and the serum was extracted and stored at −80°C for future use. The concentration of CK-MM in rat serum was determined using an ELISA kit (Jiangsu Enzyme Immunity Industry Co., Ltd.), following the manufacturer’s instructions.

### Histopathological examination

Three hours after exiting the chamber, the animals were euthanized by cervical dislocation, and suitable portions of gastrocnemius muscle and lung tissue (primarily the peripheral lung regions) were collected and fixed in 4% paraformaldehyde for 24 h. The fixed tissues were sequentially dehydrated in ethanol solutions of varying concentrations: 75% ethanol for 24 h, 85% ethanol for 2 h, 90% ethanol for 2 h, 95% ethanol for 2 h, and finally in anhydrous ethanol for 1.5 h. The tissues were then cleared using xylene for 10 min each in xylene I, II, and III. The cleared tissues were infiltrated with paraffin at 60°C: paraffin I for 1 h, paraffin II for 1 h, and paraffin III for 1 h. After paraffin infiltration, the tissues were embedded in paraffin to create wax blocks. The wax blocks were mounted on a microtome and sectioned at a thickness of 5 μm. The paraffin sections were placed in a baking machine at 60°C for 1 h. Following baking, the sections were dewaxed, rehydrated, and stained with hematoxylin and eosin (HE). Neutral resin was applied to the stained tissue sections, which were then sealed with coverslips to prevent contamination and bubbles. The slides were observed and photographed once dry.

### Observation of tissue ultrastructure

Three hours after exiting the chamber, the animals were euthanized by cervical dislocation, and a suitable amount of gastrocnemius muscle was collected and fixed in 3% glutaraldehyde. The muscle samples were then sent to the transmission electron microscopy laboratory at the Affiliated Hospital of Guangdong Medical University for embedding and sectioning. Finally, the samples were observed and photographed using an electron microscope (JEM-1010).

### Western blot analysis for protein expression

After decompression, the animals were euthanized by cervical dislocation, and an appropriate amount of gastrocnemius muscle was collected. The muscle was washed with cold saline to remove blood and hair. RIPA lysis buffer was then added, and the mixture was placed in a tissue freezing grinder for processing at −35°C. The resulting homogenate was lysed on ice for 2 h, with ultrasonic lysis performed three times during this period to ensure complete lysis. After lysis, the sample was centrifuged at 2,000 rpm for 30 min, and the supernatant was collected. Protein concentration was determined using the bicinchoninic acid (BCA) assay. Subsequently, the expression levels of p38, ERK, MyD88, and TLR9 in skeletal muscle were detected using techniques such as gel electrophoresis, immunoblotting, and chemiluminescence, with β-tubulin serving as the internal reference protein. This process was repeated three times (5).

### Real-time PCR

Total RNA was isolated from the gastrocnemius muscle using the RNeasy kit (Qiagen) and reverse transcribed with the ThermoScript RT-PCR system (Invitrogen). Real-time PCR was conducted for rat Atrogen-1, MuRF-1, and GAPDH using TaqMan Gene Expression Assays (Applied Biosystems). Relative expression levels were determined using the 2^−ΔΔCT^ method. Figure 3.7 presents data from the same set of experiments, normalized to 2^−ΔCT^.

### Statistical methods

The data statistical analysis was performed using GraphPad Prism 9. Descriptive statistics for continuous variables were presented as mean ± standard deviation ( $\bar{X}$ *±S*). One-way ANOVA was employed to compare different treatment groups, followed by Tukey’s *post hoc* test for between-group comparisons. A significance level of *p* < 0.05 was considered statistically significant.

## Results

### Incidence rate and mortality rate

Within 30 min of exiting the decompression chamber, rats exhibited symptoms of decompression sickness, including difficulty walking, hind limb paralysis, convulsions, death, tachypnea, dyspnea, and cyanosis of the extremities. As depicted in Table 1, on the first day, 7 (22%) of the 32 rats in the decompression group died, and autopsy results confirmed pulmonary embolism as the cause. Consequently, a total of 41 rats were included in the anatomical analysis of serum, skeletal muscle, and lungs for this study. Other symptoms, such as difficulty walking (44%), quadriplegia (6%), dyspnea (32%), and cyanosis (32%), indicate severe decompression sickness. However, as shown in Table 1, the mortality rate of rats gradually decreased with the number of decompressions.

**Table 1:** Observation results of symptoms in each group of rats.

Grouping			Behavioral indicators			Respiratory indicators		
			Difficulty in walking	Limb paralysis	Death	Rapid breathing	Difficulty breathing	Cyanosis
Control group			0/8 (0%)	0/8 (0%)	0/8 (0%)	0/8 (0%)	0/8(0%)	0/8(0%)
Normal pressure group			0/8 (0%)	0/8 (0%)	0/8 (0%)	0/8 (0%)	0/8(0%)	0/8(0%)
Decompression group	Day 1	D1 group	5/8 (63%)	1/8 (13%)	2/8 (25%)	8/8 (100%)	2/8(25%)	3/8(38%)
		D2 group	2/8 (25%)	0/8 (0%)	2/8 (25%)	7/8 (88%)	3/8(38%)	3/8(38%)
		D3 group	3/8 (38%)	0/8 (0%)	2/8 (25%)	6/8 (75%)	3/8(38%)	3/8(38%)
		D4 group	4/8 (50%)	1/8 (13%)	1/8 (13%)	6/8 (75%)	2/8(25%)	1/8(13%)
	Day 2	D2 group	2/6 (33%)	0/6 (0%)	0/6 (0%)	6/6 (100%)	1/6(17%)	1/6(17%)
		D3 group	1/6 (17%)	1/6 (17%)	1/6 (17%)	6/6 (100%)	2/6(33%)	1/6(17%)
		D4 group	3/7 (43%)	0/7 (0%)	1/7 (14%)	5/7 (71%)	1/7(14%)	2/7(29%)
	Day 3	D3 group	2/5 (40%)	1/5 (20%)	0/5 (0%)	5/5 (100%)	1/5(20%)	0/5(0%)
		D4 group	0/6 (0%)	0/6 (0%)	0/6 (0%)	6/6 (100%)	0/6(0%)	0/6(0%)
	Day 4	D4 group	0/6 (0%)	0/6 (0%)	0/6 (0%)	6/6 (100%)	0/6(0%)	0/6(0%)

### Vascular bubbles

**Figure 2:**
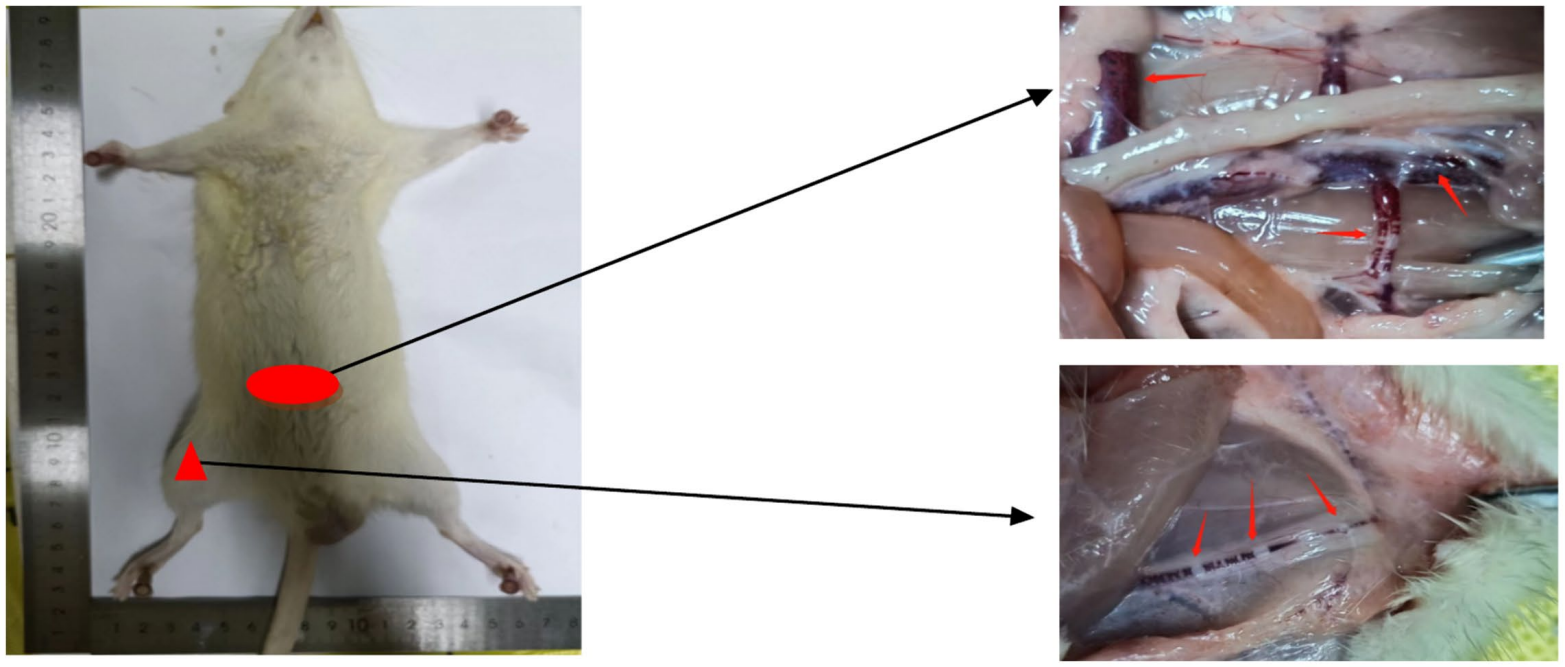
Bubble diagram of blood vessels in rats that died after decompression. Macroscopic injuries in decompression sickness deceased rats. Examination revealed the presence of macroscopic bubbles in the femoral vein (indicated by triangular markers) and mesenteric vein (indicated by elliptical markers) in deceased rats.

As shown in Figure 2, a significant number of bubbles or filled blood vessel sections can be clearly observed in the subcutaneous veins, femoral veins, and mesenteric veins of rats that died after exiting the chamber. These bubbles were also widely distributed throughout other blood vessels. In contrast, no visible bubble formation was found in the bodies of the surviving rats. This observation suggests a direct correlation between bubble formation and rat mortality.

### Pulmonary tissue structural changes

**Figure 3:**
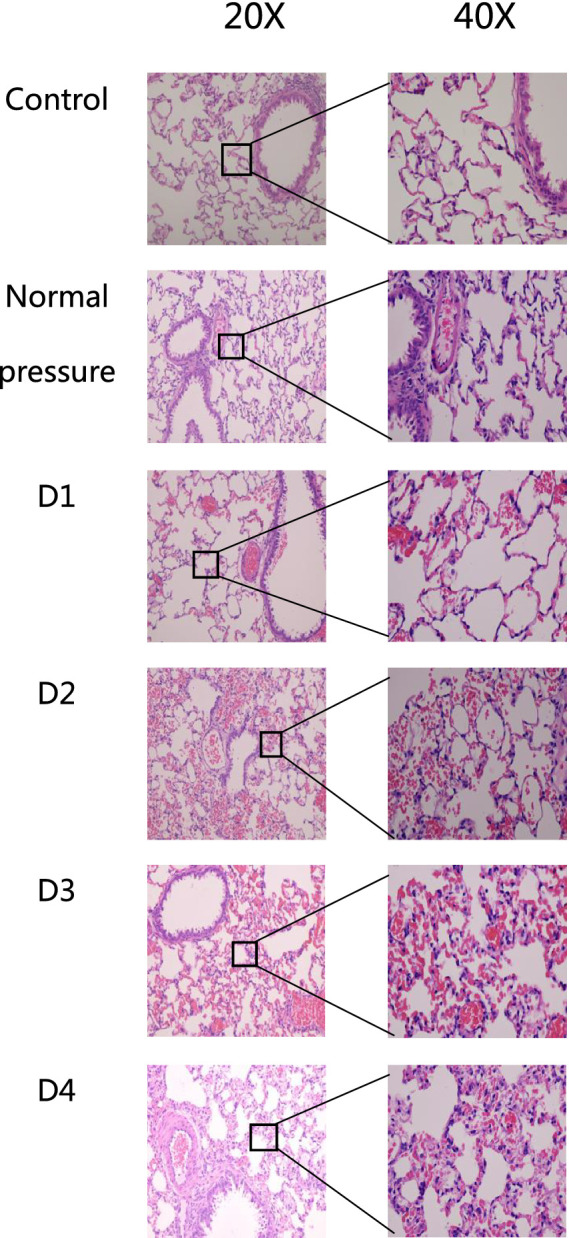
HE staining of rat lung tissue in each group. After one decompression event (D1), there is minimal pulmonary bleeding in rat lung tissue. Following two decompressions (D2), the amount of alveolar hemorrhage significantly increases. After three decompressions (D3), rat lung tissue shows signs of inflammatory cell infiltration, with partial absorption of blood cells. Comparing the fourth decompression (D4) to the third (D3), there are no significant differences observed in lesion severity.

The sampling time, as depicted in Figure 3, was 3 h after decompression. After a single decompression, the structure of the rat lung tissue did not show significant changes. However, repeated decompressions caused marked lung tissue structural disorder. After one decompression, slight hemorrhaging began in the lung tissue. Following a second decompression, the number of alveolar red blood cells increased significantly. After a third decompression, inflammatory cell infiltration appeared in the lung tissue, and some blood cells were partially absorbed. There was no significant difference in the lesions between the third and fourth decompressions, suggesting that the rats may have adapted to multiple decompression treatments.

### Skeletal muscle tissue structural changes

**Figure 4:**
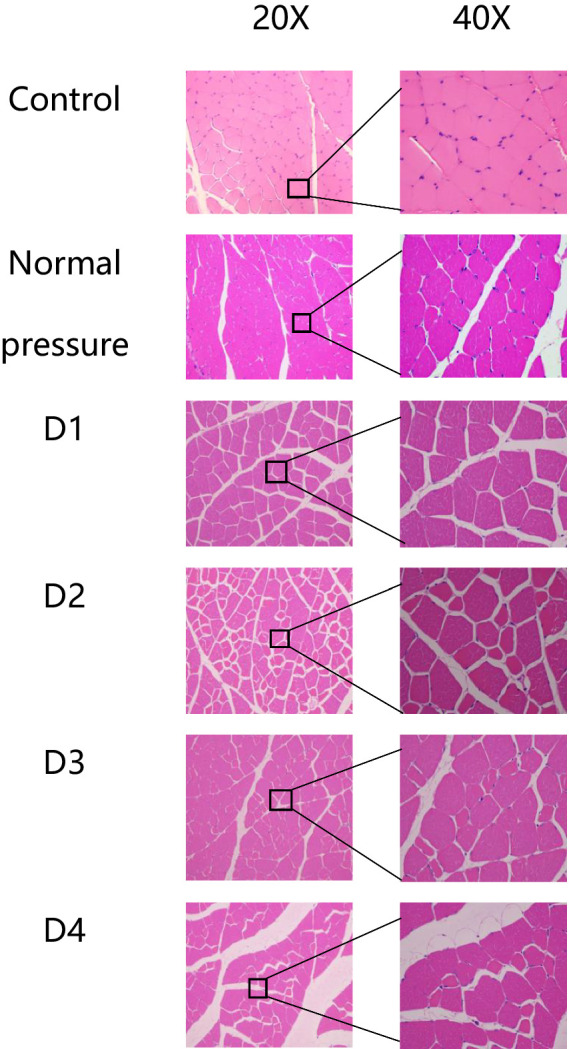
HE staining of rat muscle tissue in each group (cross-section). In the control and normal pressure groups, rat muscle fibers are neatly arranged, tightly packed, with cell nuclei close to the cell membrane. Following the first decompression event (D1), there were no significant changes observed in muscle fibers. After the second (D2), third (D3), and fourth (D4) decompressions, localized interstitial edema, muscle fiber atrophy, and disappearance of cell nuclei were observed.

**Figure 5:**
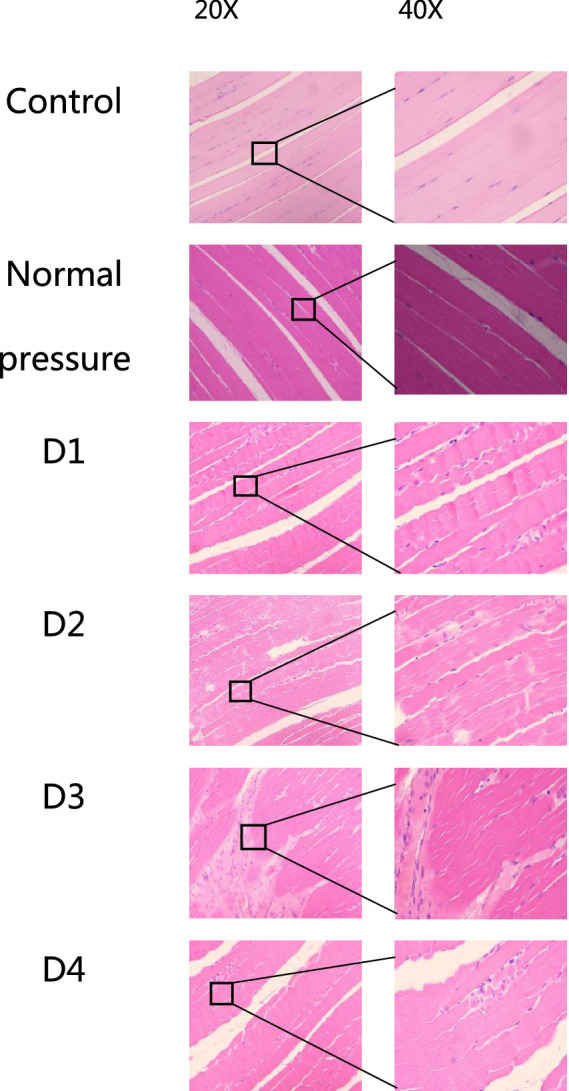
HE staining of rat muscle tissue in each group (longitudinal section). In the control and normal pressure groups, rat muscle fibers are neatly arranged, tightly packed, with cell nuclei close to the cell membrane. After the first (D1), second (D2), third (D3), and fourth (D4) decompressions, localized muscle fiber fragmentation and waviness were observed.

The following six groups of images were obtained from the blank control group, the normal pressure group, and rats at various stages of decompression. All samples were collected 3 h after decompression treatment, as depicted in Figures 4, 5. Following a single decompression treatment, we observed no significant changes in the skeletal muscle tissue structure of the rats. However, with repeated decompression treatments, the skeletal muscle tissue structure of the rats exhibited significant disorder. In the control and normal pressure groups, we observed neatly arranged muscle fibers with closely positioned cell nuclei near the cell membrane. In rats subjected to a single decompression treatment, their muscle fibers showed no significant changes. However, with successive decompression treatments—second, third, and fourth times—local interstitial edema, muscle fiber atrophy, and even the disappearance of cell nuclei gradually became evident. It is noteworthy that no significant differences were observed when comparing the pathological conditions following the third and fourth decompression treatments. This suggests the possibility of an adaptive phenomenon in rats following repeated decompression treatments.

### Skeletal muscle ultrastructural changes

**Figure 6:**
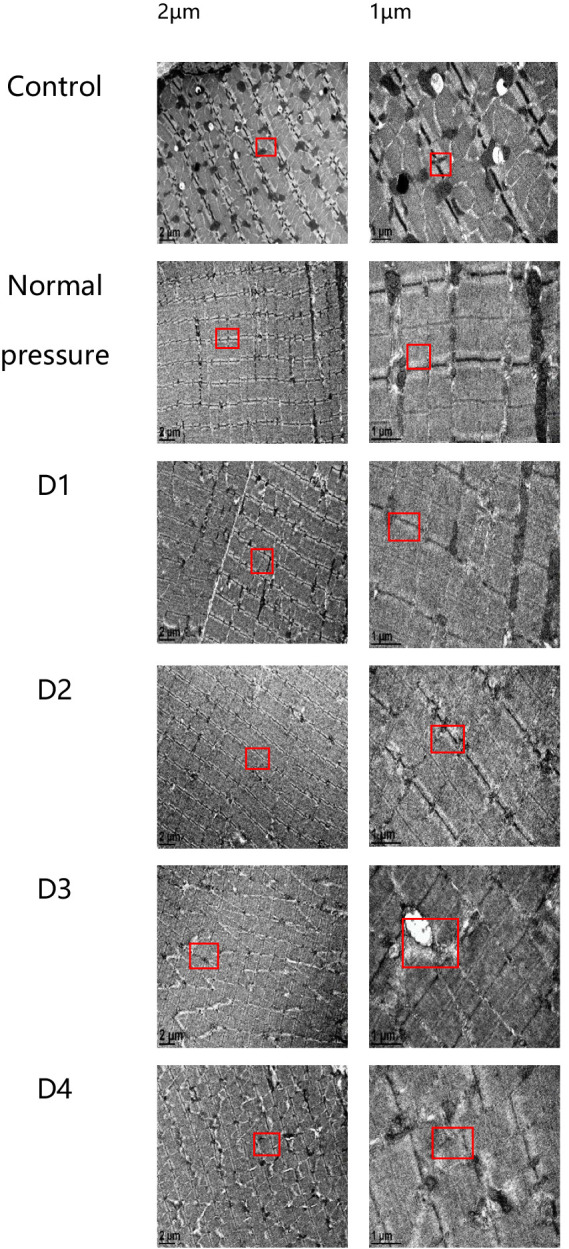
Electron microscope sections of rat muscle tissue in each group. In the control and normal pressure groups, rat skeletal muscle myofibrils are neatly arranged, with clear regularity and repeatability of muscle segments. Mitochondria vary in size and are evenly distributed on both sides of the Z-lines, with some also distributed beneath the muscle membrane. They predominantly exhibit elongated or oval shapes, enclosed by double membranes, and their internal cristae structures are clearly visible. Following the first (D1), second (D2), third (D3), and fourth (D4) decompressions, localized disruptions in muscle segment structure gradually appear, occasionally accompanied by myofilament fractures, and some mitochondria exhibit swelling or detachment of the inner membrane.

The following six sets of images represent the blank control group, the normal pressure group, and rats subjected to various decompression treatments. All images were captured 3 h after decompression treatment, as depicted in Figure 6. Following a single decompression treatment, the ultrastructure of rat muscle tissue did not exhibit significant changes. However, following repeated decompression treatments, the ultrastructure of their muscle tissue exhibited significant disorder. In both the control group and the normal pressure group, we observed that the myofibrils of rat skeletal muscles were arranged neatly, with clear regularity and repetitiveness in the sarcomeres. The size of the mitochondria was variable, yet they were evenly distributed on either side of the Z line, with some located beneath the sarcolemma. Most mitochondria exhibited a three-dimensional elongated or ovoid shape, enveloped by double membranes, and displaying clearly visible cristae structures inside. However, with an increasing number of decompression sessions, we progressively observed disordered local sarcomere structures, occasionally noting myofilament ruptures. Additionally, some mitochondria displayed structural abnormalities, including swelling and detachment of the inner membrane. These findings suggest that repeated decompression treatments can significantly impact the ultrastructure of rat muscle tissue.

### Decompression sickness triggers an increase in the expression of inflammatory factors in muscle tissue

**Figure 7:**
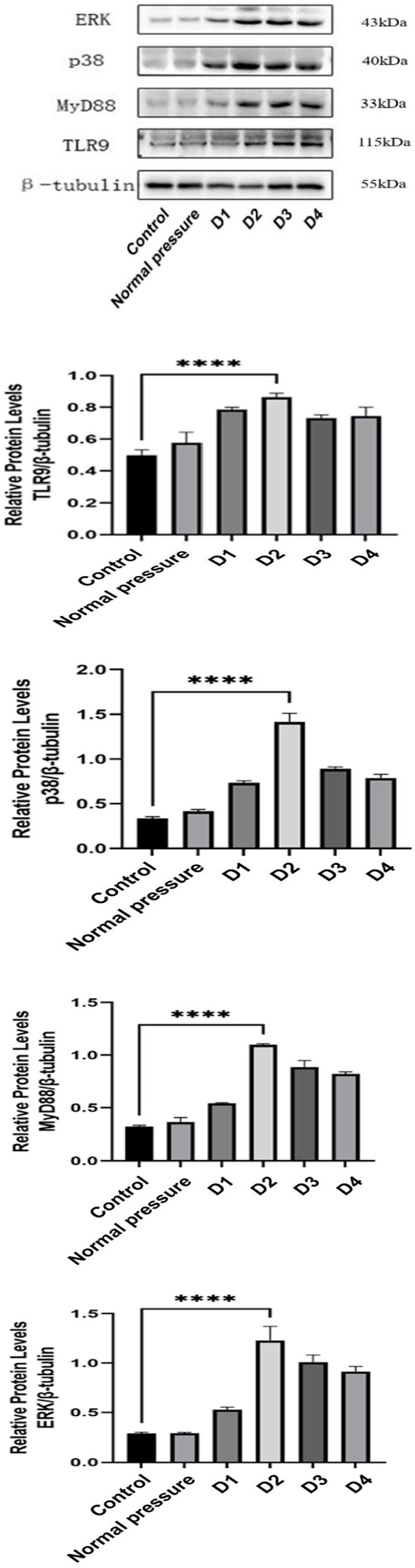
Comparison of TLR9, MyD88, ERK, and p38 protein levels in skeletal muscle of rats in different groups.

Decompression sickness can induce elevated expression levels of TLR9, MyD88, ERK, and p38 proteins in skeletal muscle tissue, as clearly depicted in Figure 7. Compared to the blank control group and the normal pressure group, the experimental group showed a significant increase in the expression of TLR9, MyD88, ERK, and p38 proteins with successive decompression treatments. Further observation revealed that the D3 and D4 groups exhibited a slight decrease compared to the D2 group, albeit not reaching statistical significance (*p* > 0.05). This trend may suggest that rats develop an adaptive mechanism following multiple decompression treatments. In summary, improper decompression procedures can significantly activate the TLR9-MyD88 signaling pathway in skeletal muscle tissue, leading to increased expression levels of related proteins. The stable or slightly decreased protein expression after repeated decompressions likely reflects the adaptation process of rats to decompression stress.

### Decompression sickness induces the expression of Atrogin-1 and MuRF-1 mRNA in rat skeletal muscle

**Figure 8:**
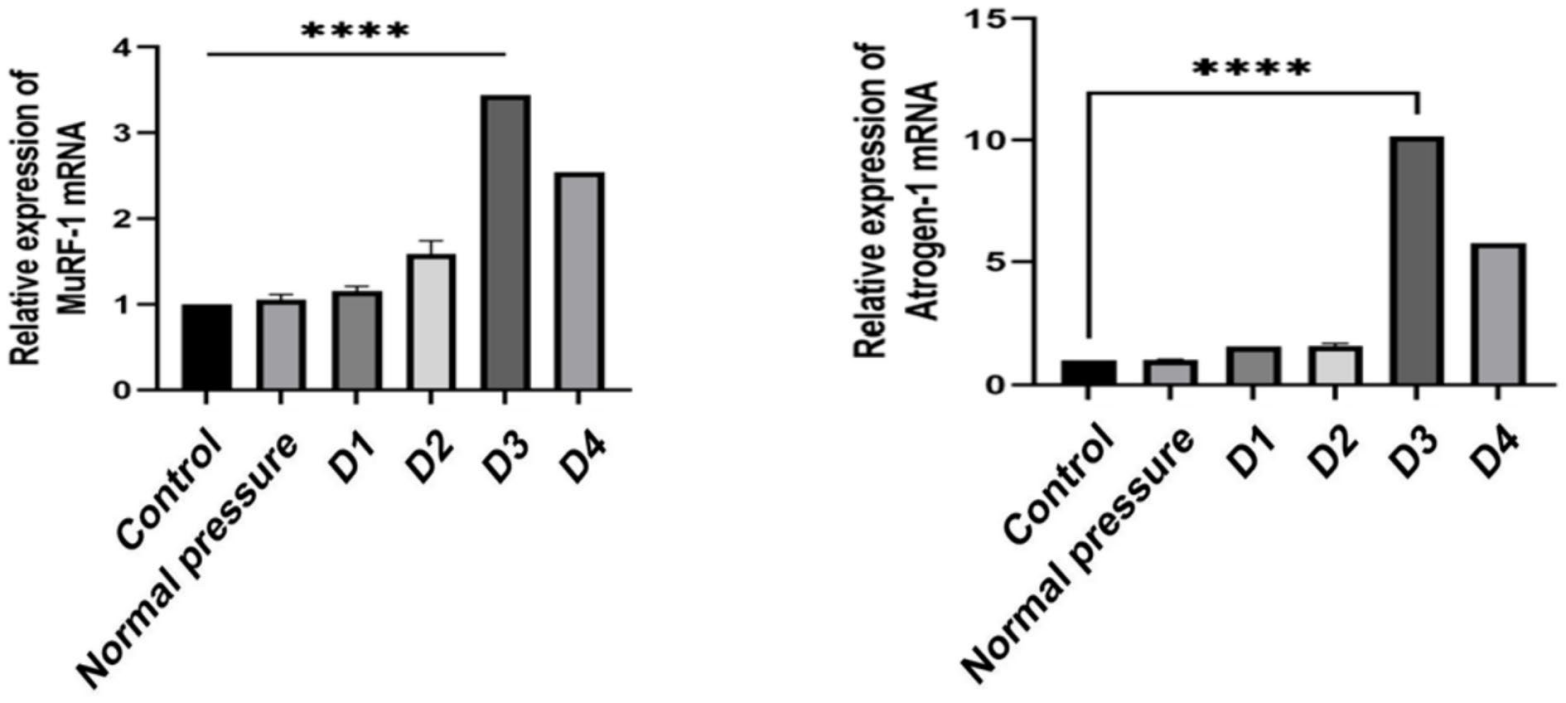
Comparison of Atrogin-1 and MuRF-1 mRNA expression levels in skeletal muscle of rats in different groups.

Based on the data presented in Figure 8, we observed that the D1 and D2 groups did not exhibit significant changes in the expression of Atrogin-1 mRNA and MuRF-1 mRNA compared to the control and normal pressure groups. However, with an increasing number of decompressions, we noted a gradual rise in the expression levels of Atrogin-1 mRNA and MuRF-1 mRNA. This suggests that repeated decompression treatments may induce a trend toward muscle tissue atrophy.

### Content of CK-MM

**Figure 9:**
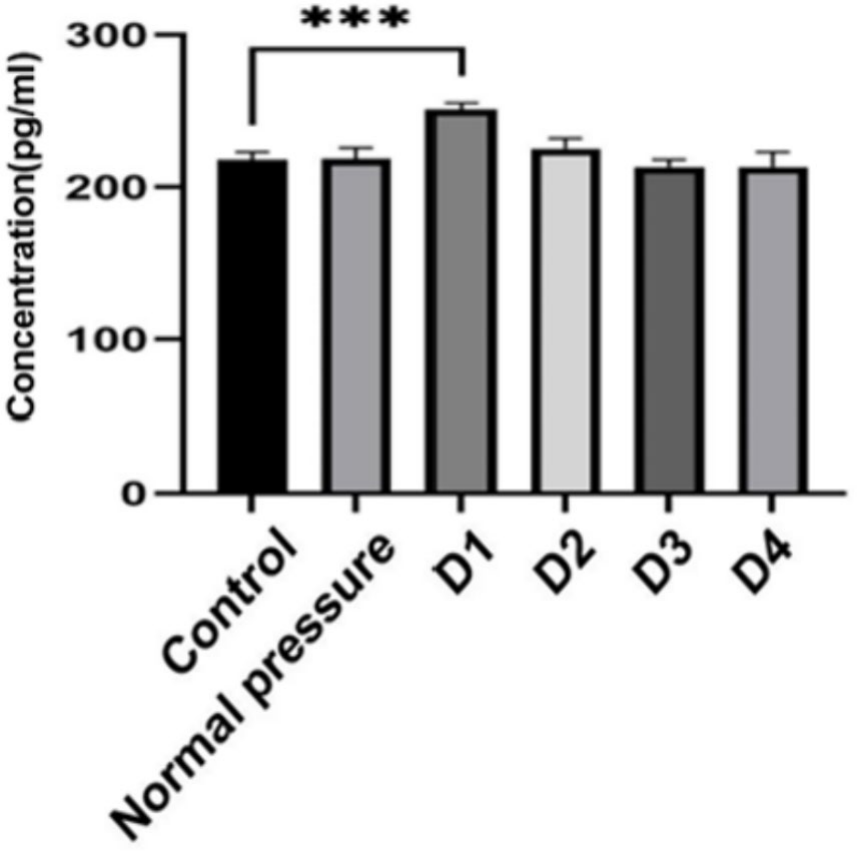
Changes in CK-MM content. ^***^indicates statistically significant differences between groups (*p* < 0.05).

Based on the data analysis from Table 2 and Figure 9, significant differences were observed between group D1 and both the control group and the normal pressure group. Specifically, group D1 exhibited a statistically significant increasing trend (*p* < 0.05). In contrast, groups D2, D3, and D4 showed a decreasing trend compared to group D1, and these decreases were also statistically significant (*p* < 0.05). It is noteworthy that while there were numerical differences between groups D3 and D4, these distinctions did not reach statistical significance (*p* > 0.05). These findings suggest that after repeated decompression treatments, skeletal muscle tissue may experience transient damage, with the most pronounced effects occurring during the initial phases of decompression.

**Table 2:** The CK-MM concentration (pg/mL) at different time points for rats in each group (X̅±S).

Grouping	CK-MM concentration (pg/mL)
Control	218.13 ± 5.02
Normal pressure	218.74 ± 7.23
D1	251.20 ± 4.13
D2	225.35 ± 6.75
D3	213.31 ± 4.93
D4	213.47 ± 9.61

## Discussion

As diving has evolved from its military and commercial roots to a popular recreational pursuit, health professionals have increasingly turned their attention to the diverse health risks associated with diving (6). Early studies indicate that changes in environmental pressure, leading to the buildup of gas bubbles—primarily nitrogen—within bodily tissues, constitute the primary mechanism contributing to tissue damage, including necrosis (7–9). The presence of gas bubbles, viewed as exogenous entities, applies pressure on tissues, triggers an inflammatory response, and can induce tissue hypoxia. The dispersion of these gases at different rates throughout the body leads to systemic changes. Live imaging of rats post-decompression revealed visible small bubbles in tissues such as subcutaneous fat, subcutaneous veins, and lower limb veins. Previous research has shown that around 30 min after decompression, the quantity of bubbles within experimental subjects peaks (10). The effects of post-decompression bubbles can range from mild discomfort or sensory abnormalities to more severe outcomes such as paralysis and death. Presently, a substantial portion of diving research concentrates on cardiovascular conditions, including disorders of the pulmonary, neurological, and otolaryngological systems (6).

This study employed a rat model of decompression sickness to evaluate its efficacy. A critical aspect of the investigation was the observation of symptoms and signs in the rats upon exiting the decompression chamber, including fur erection, skin itching, seizures, decreased activity, and 100% mortality. In the realm of decompression sickness research, mortality is a commonly observed and unpredictable outcome (11, 12). The findings of this study indicate that the mortality rate following the initial decompression event in rats is 22%. However, as the frequency of decompression events increases, the mortality rate progressively decreases, suggesting an adaptive response of rats to the physiological damage caused by decompression. An analysis of 40,000 caisson workers undergoing decompression revealed that the incidence of decompression sickness decreased from approximately 12 to 1% within the initial 10–15 daily decompression sessions (5 days per week). This suggests that repeated exposure to increased pressure may confer some protection against DCS (13, 14). This physiological phenomenon of protection from repeated or prolonged exposure to compressed air is termed adaptation. Research findings suggest that repeated exposure to compression-decompression stress can induce the expression of genes related to stress and inflammation, aiding rats in adapting to rapid decompression (15). Another aspect of the study involved observing morphological changes in visceral organs, with particular attention to the lungs as critical target organs for bubbles formed in the venous system (16–20). The pathological results of rat lung tissues in this study indicated acute lung injury (ALI), characterized mainly by red blood cell aggregation in lung tissue blood vessels and pulmonary interstitial edema, which worsened with repeated improper decompression. This finding is consistent with previous research (21).

Skeletal muscle injuries often result from trauma-induced contusions or tears, leading to reduced muscle function. Additionally, exposure to extreme temperatures, toxins, and other acute insults can also cause damage. Human tissues possess a degree of adaptability, enabling adjustments in gene transcription levels and protein stability to accommodate environmental fluctuations. However, if environmental stressors exceed the adaptability threshold, tissue damage and potential necrosis may ensue (22). This study is based on the hypothesis that muscle tissue sustains damage under conditions of extreme air pressure. Additionally, previous research has identified muscle fiber degeneration and necrosis in the skeletal muscles of marine mammals following decompression (3). Decompression sickness is currently categorized into two types: Type 1, characterized by mild pain or mild skin symptoms, and Type 2, which includes neurological complications. Injuries to the musculoskeletal system, such as femoral head necrosis and flexor pain associated with muscle damage, are also frequently observed (23). Improper decompression results in the release of excessive inert gas into adjacent tissues, predominantly nitrogen escaping from solution. This process subsequently forms bubbles within tissues and blood, thereby inducing inflammatory stress on blood vessels, nerves, and tendons (24, 25). This study provides evidence of muscle tissue damage in response to decompression sickness conditions, as indicated by observed morphological changes such as fiber rupture and distortion, as well as subtle modifications in ultrastructure, including alterations in mitochondria and muscle sarcomeres. Moreover, serum markers such as creatine kinase, which are degradation products of muscle fiber damage, serve as precise indicators of the severity of muscle injury. Elevated serum creatine kinase (CK-MM) levels indicate more severe skeletal muscle damage (26). Our study revealed transient increases in specific markers of muscle damage following improper decompression. Collectively, these empirical findings suggest that decompression sickness has the potential to induce muscle injury.

TLR9, MyD88, ERK, and p38 proteins are critical inflammation-related molecules. They are not only closely associated with the regulation of inflammatory gene expression but also play a significant role in the activation of inflammatory cells. Studies have shown that skeletal muscle mitochondrial fragmentation can promote TLR9-dependent inflammatory responses, exacerbating muscle atrophy and leading to an overall decline in physical function. The electron microscopy images of muscle tissue in this study also revealed mitochondrial structural damage following improper decompression. Moreover, Rongjie Zhou et al. (27) have demonstrated that the TLR9/MyD88 signaling pathway is implicated in regulating the inflammatory response associated with exercise-induced skeletal muscle injury and is a primary contributor to delayed onset skeletal muscle soreness. Studies have demonstrated that activation of the Toll-like receptor (TLR)-MyD88 pathway can enhance protection against decompression sickness (28). Bigley et al. (2) conducted a study to analyze blood and tissue biomarkers as potential indicators for early detection of decompression sickness. They observed changes in inflammatory mediators within a 24-h timeframe following decompression in female Sprague–Dawley rats. It was found that levels of inflammatory cytokines, including TNF-alpha, IL-6, and IFN-gamma, increased in the blood 6 h after decompression. Expression of ICAM-1, E-selectin, and L-selectin in blood vessels and tissues also significantly increased 24 h after decompression, indicating that rapid decompression triggers the release of inflammatory mediators and leads to tissue inflammation. This study demonstrates a transient increase in the expression of TLR9, MyD88, ERK, and p38 proteins in muscle tissue following improper decompression, indicating a significant association between this injury process and the inflammatory response. Further investigation into the regulatory mechanisms of muscle tissue injury and potential treatment strategies is crucial for enhancing our understanding of decompression-induced muscle injury mechanisms and identifying novel targets and approaches for managing associated conditions.

Atrogin-1 and MuRF-1 are pivotal regulatory proteins linked to muscle atrophy, playing critical roles in its pathogenesis. During conditions of muscle atrophy, such as prolonged immobilization, sedentary lifestyle, persistent inflammation, and muscle inactivity, there is notable upregulation of Atrogin-1 and MuRF-1 expression levels (29–31). Therefore, excessive expression of Atrogin-1 and MuRF-1 is considered a significant molecular marker for muscle atrophy. This study observed a transient increase in the expression of these genes with the accumulation of improper decompression, indicating that decompression-related injuries can indeed contribute to muscle atrophy.

## Conclusion

In the experimental conditions of this study, it was observed that a single decompression event had minimal impact on muscle tissue architecture. However, repeated decompression episodes led to detectable muscle tissue damage, although the severity of this damage appeared to stabilize with prolonged exposure. This adaptation may be attributed to the resilience of the rat model and the duration of the study’s observation period. It is crucial to recognize that while rats share structural and functional similarities with human cardiovascular systems, there may be discrepancies in their physiological responses to decompression. Therefore, the study’s findings may have limitations in fully replicating human conditions. To enhance the assessment of decompression’s impact on human muscle tissue, future research should aim to include a larger cohort of experimental subjects and integrate clinical data for further validation.

## Data availability statement

Datasets are available on request: The raw data supporting the conclusions of this article will be made available by the authors, without undue reservation.

## Ethics statement

The animal study was approved by Laboratory Animal Ethics Committee of the Affiliated Hospital of Guangdong Medical University/Affiliated Hospital of Guangdong Medical University. The study was conducted in accordance with the local legislation and institutional requirements.

## Author contributions

GC: Writing – original draft, Writing – review & editing, Funding acquisition. YH: Writing – original draft, Writing – review & editing. CH: Writing – original draft, Data curation. LL: Writing – original draft, Writing – review & editing, Data curation, Validation. JP: Writing – original draft, Writing – review & editing, Investigation, Methodology. HL: Writing – original draft, Writing – review & editing, Investigation, Methodology. WZ: Writing – original draft, Writing – review & editing, Investigation, Methodology.
